# Cecal Volvulus Post Cesarean Section: A Case Report

**DOI:** 10.7759/cureus.6644

**Published:** 2020-01-13

**Authors:** Mohammed Basendowah, Mutaz H Alabdulqader, Ossamah Abdulqader, Muatasaim Hakami

**Affiliations:** 1 Surgery, King Abdulaziz University Hospital, Jeddah, SAU

**Keywords:** cecal volvulus, volvulus, large bowel obstruction, exploratory laparotomy

## Abstract

Cecal volvulus is a very rare cause of large bowel obstruction (LBO) that develops when a part of the bowel twists around the mesentery. Cases of acute abdomen, regardless of age, race, and ethnicity, should be examined to exclude volvulus from differential diagnoses. Surgery is the only confirmatory method to diagnose and treat this life-threatening condition. Here, we report a case of a 35-year-old female patient who presented with abdominal pain, distension, constipation, and vomiting. Abdominal computed tomography (CT) aided in accurately diagnosing the cecal volvulus, and the patient immediately underwent an exploratory laparotomy.

## Introduction

Volvulus is diagnosed when a part of the bowel twists around its mesentery [1]. Colonic volvulus is a type of large bowel obstruction (LBO) that may present with abdominal pain, abdominal distension, and decreased or no passage of stool and gases [2]. Volvulus accounts for 10%-15% of the cases of LBO, with sigmoid volvulus being the most common type accounting for 80% of all volvulus cases, while cecal volvulus represents 15% of such cases [1]. The main etiology of volvulus is related to midgut development in an embryo wherein failure of intestinal rotation may cause fixation of the mesentery to the cecum, leading to a high risk of cecal volvulus in the future [3]. Here, we describe a case of a cecal volvulus that was diagnosed and managed at the King Abdulaziz University Hospital in Jeddah.

## Case presentation

A 35-year-old female patient with a previous history of three cesarean sections and known gastroesophageal reflux disease, taking proton pump inhibitors, presented to the emergency room in our hospital with abdominal distension mainly on the right side for six days associated with abdominal pain, constipation, and no passage of gases. She also complained of nausea and vomiting after eating or drinking. The patient had sought medical advice twice in the outpatient clinic due to constipation and was discharged with a prescription of laxatives; however, no improvement in the symptoms was noted by the patient.

Physical examination, upon arrival to the emergency room, revealed that the patient was tachycardic (127 beats per minute) and afebrile (36.3°C) with an oxygen saturation of 100% in room air, slightly elevated respiratory rate at 24 breaths per minute, and normal blood pressure (136/88 mmHg). On general examination, the patient was found to be alert. The abdomen was hugely distended mainly on the right side with abdominal tenderness overall; however, there was more localized tenderness on the right side and very sluggish bowel sounds. Digital rectal examination revealed an empty rectum and no incisional or ventral hernia.

Hematological tests revealed anemia (hemoglobin level 8 g/dl), with a mean cell volume of 60.6 fL and mean cell hemoglobin level of 18.2 pg. Elevated platelet count at 666,000 per mL/L with no leukocytosis was noted. Biochemical investigations showed low sodium (128 mmol/L; normal, 136-145 mmol/L), low potassium (3.1 mmol/L; normal, 3.5-5.5 mmol/L), low chloride (88 mmol/L; normal, 98-107 mmol/L), high urea (16.9 mg/dL; normal 2.5-6.4 mg/dL), slightly high creatinine (140 µmol/L; normal, 53-115 µmol/L), and normal lactic acid (0.7mmol/L; normal, 0.4-2.0 mmol/L) levels. The patient had a normal coagulation profile.

**Figure 1:**
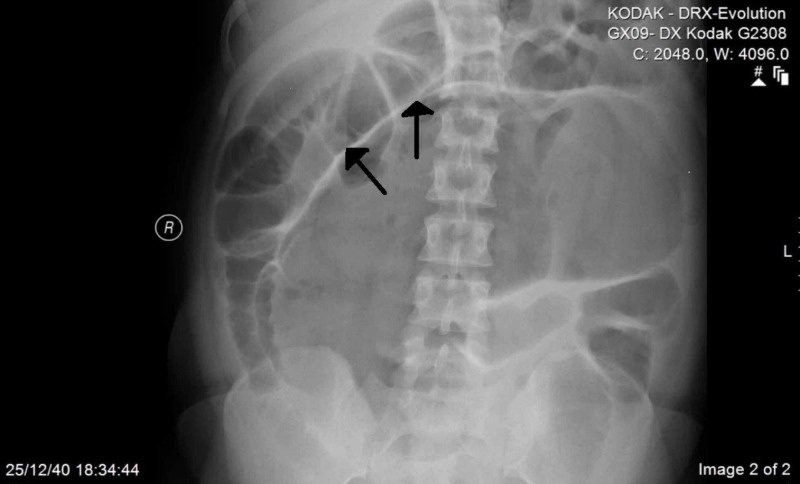
Abdominal radiograph taken in the supine position showing the dilated cecum from the right lower quadrant to the left upper quadrant and the air-fluid level

An abdominal radiograph showed a markedly distended bowel loop on the right side and at the mid-line with dilated small bowel loops suggestive of cecal volvulus (Figure 1).

**Figure 2:**
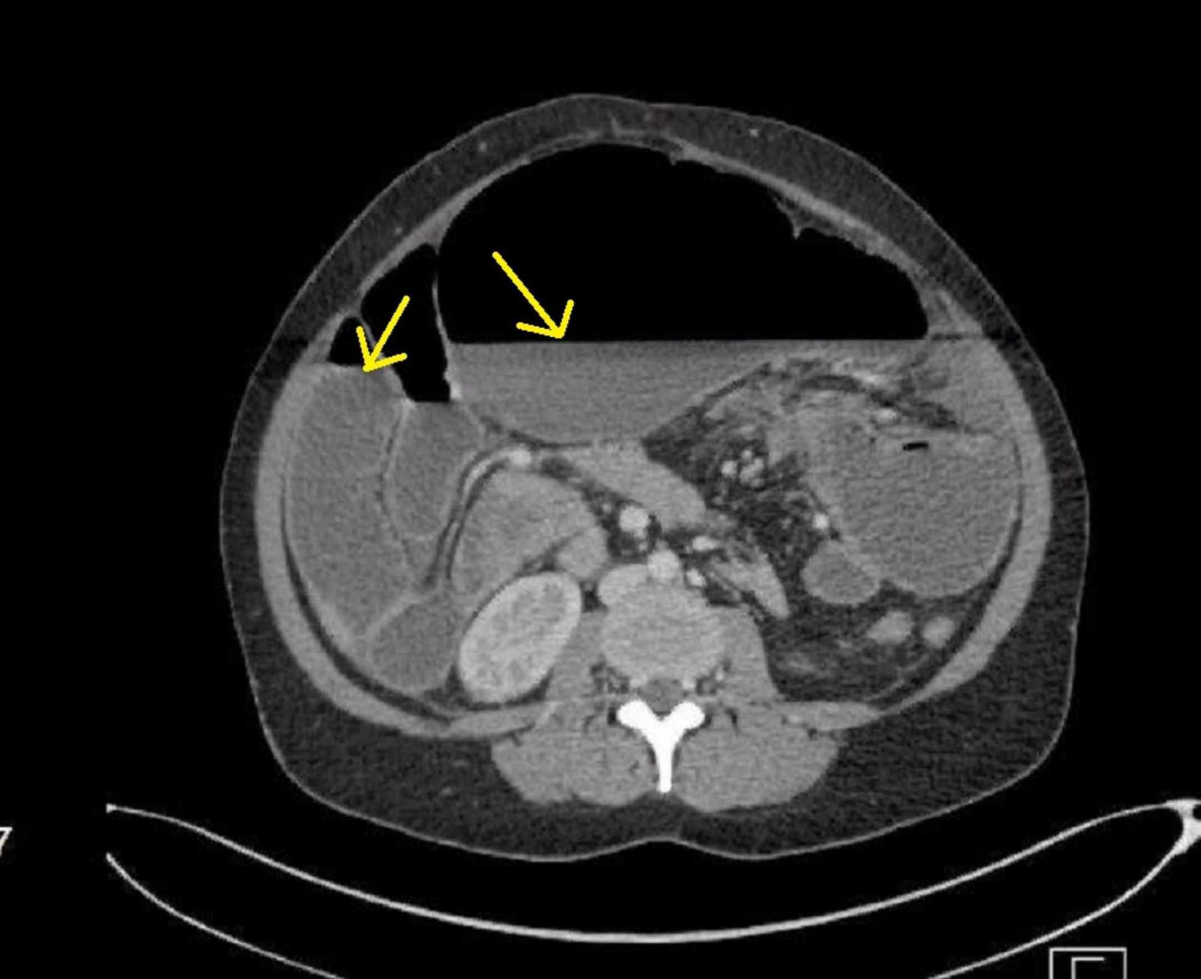
Abdominal computed tomography with contrast revealing the dilated bowel loop and air-fluid

Abdominal computed tomography (CT) showed markedly dilated cecum with air-fluid appearing upside down and a mesenteric twist being indicated by the twisting mesenteric vessels. The mural enhancement of the bowel loops was preserved, and no free air was noted. These image findings suggest cecal volvulus with complete LBO with no evidence of perforation (Figure 2).

**Figure 3:**
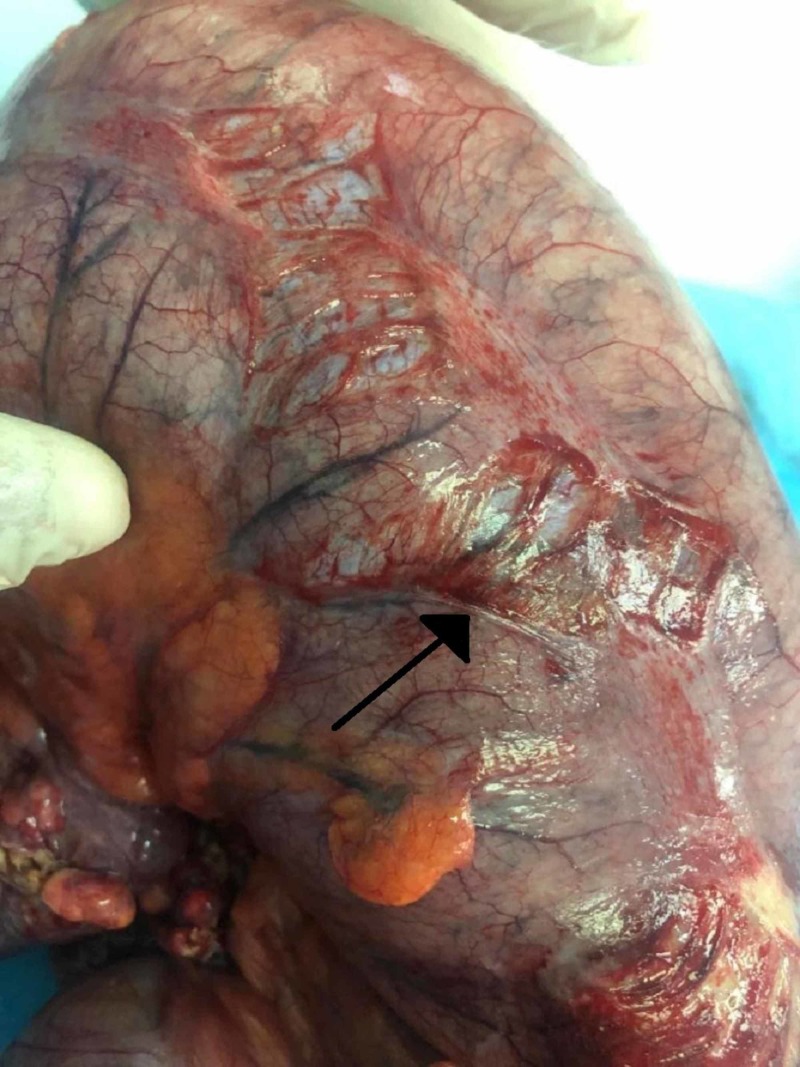
Serosal tear indicating an impending perforation of the colon

After resuscitating the patient in the emergency room, an urgent exploratory laparotomy was performed. During laparotomy, upon entering the abdomen, a massively dilated cecum, dilated proximal small bowel, free intraperitoneal fluid, and a small umbilical hernia were noted. Cecal volvulus with an axial rotation was definitively diagnosed with serosal tears indicating an impending perforation (Figure 3). 

**Figure 4:**
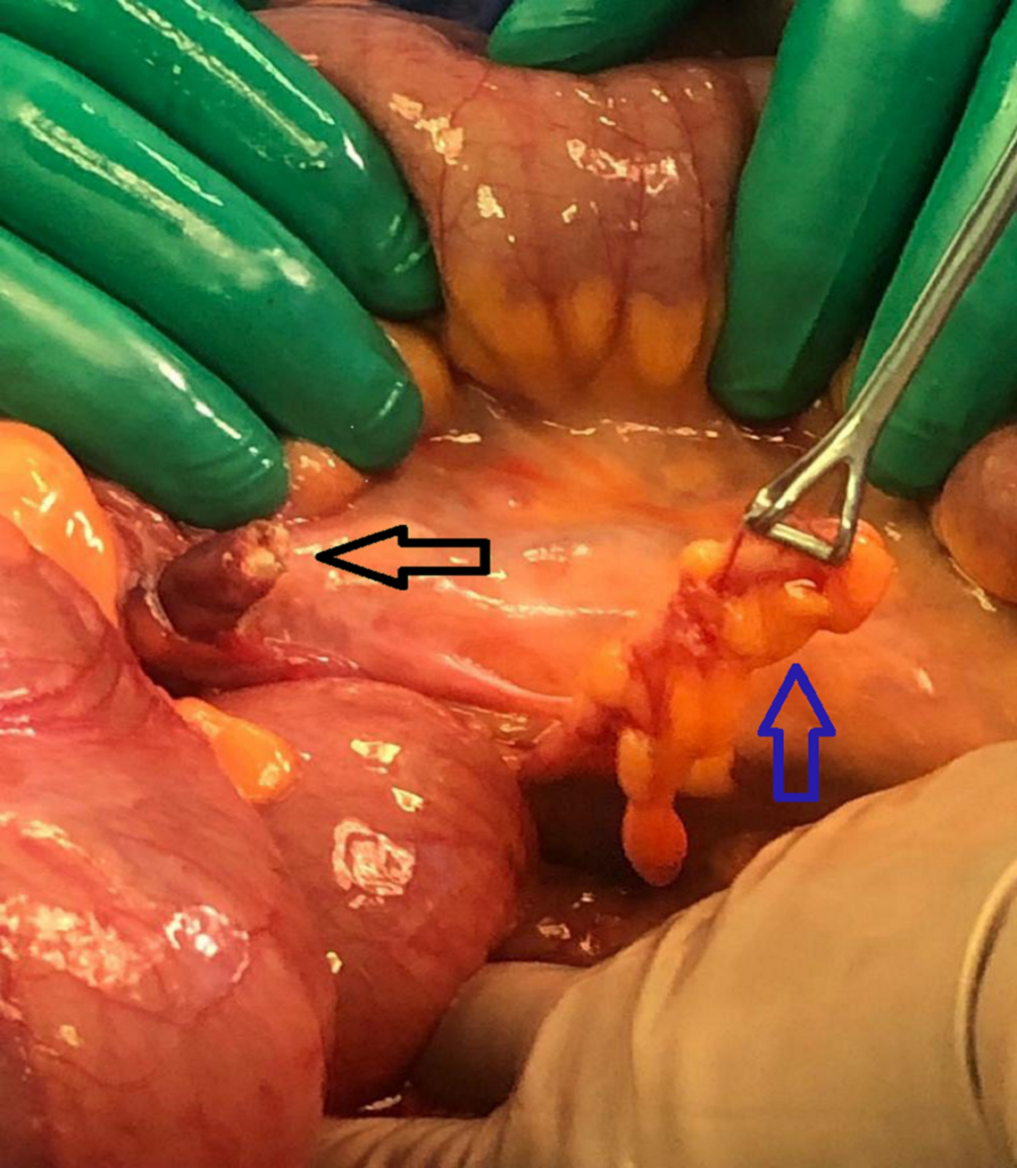
Iliocecal area Black arrow shows the band while the blue arrow indicates the appendix being held by the Babcock forceps.

Several formal bands between the ileocecal area and abdominal wall were noted and released with the Babcock forceps holding the appendix while the thick band was released. Subsequently, the ileocecal region was rotated to its normal anatomical position (Figure 4).

Careful rotation of the cecum and dissection of the tissue around the ileocecal valve were performed to expose the transverse colon and terminal ileum. Right hemicolectomy and side-to-side anastomosis with a 55-mm gastrointestinal anastomosis stapler were performed. The edges were cut using a transverse anastomosis, and the abdomen was closed.

After the surgery, the patient was taken to the surgical intensive-care unit and extubated eight hours after the surgery. She was then shifted to the intermediate care unit for two days for pain management and cardiorespiratory monitoring. The patient tolerated feeding on post-operative day two and bowel motion on postoperative day three. She was followed up at the outpatient department one week after the surgery; the patient was doing well, without nausea, vomiting, or constipation. On physical examination, the laparotomy scar was clean and the abdomen was soft and lax with no incisional hernia; follow-up colonoscopy was unremarkable.

**Figure 5:**
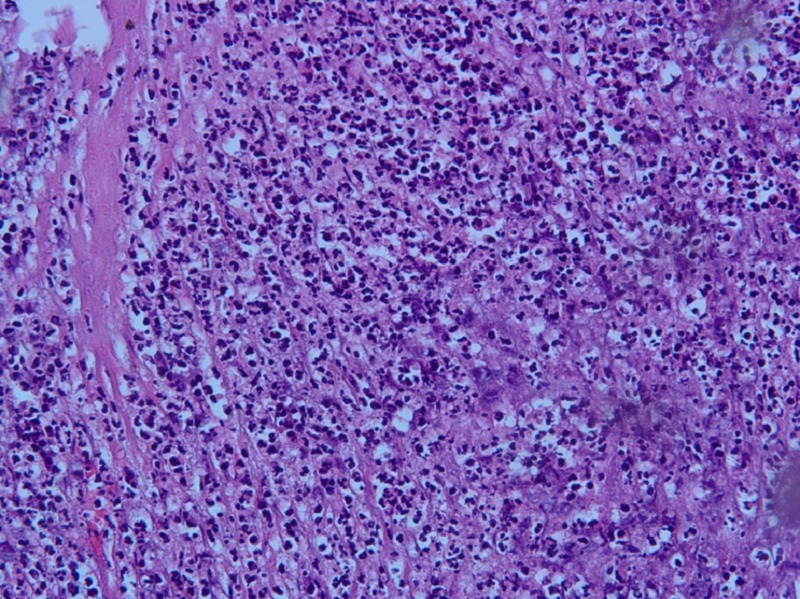
Histopathological examination showing necrotic changes in the right colon

The pathological specimen was sent for examination. The right colon revealed ischemic necrosis of the cecum consistent with a diagnosis of volvulus, while no pathological abnormality was noted in the appendix (Figure 5).

## Discussion

Volvulus is the twisting of a part of the sigmoid, cecal, or transverse colon around its mesentery [1], with sigmoid volvulus being the most common in contrast to cecal or transverse volvulus [2]. The risk factors of volvulus are equivocal and include adhesions due to past surgery, which is the most common, childbirth, a redundant and freely movable cecum, and left colon obstruction owing to different reasons. A volvulus rotates around its axis, and thus, is prone to precipitate vascular strangulation and ischemic changes in the mesentery. Patients with a volvulus typically present to the emergency room with various non-specific gastrointestinal symptoms, including abdominal pain, abdominal distension, vomiting, or constipation, all of which indicate LBO, but with no exact cause. Physical examination findings also vary considerably with abdominal tenderness and distension being the most common to the presentation of a mass in the abdomen in some cases [4-5]. Laboratory investigations are not sufficiently helpful in cases of cecal volvulus [6]. By contrast, radiographic imaging, including abdominal radiography and CT, could confirm the diagnosis in up to 90% of volvulus cases [7].

Surgical intervention provides the most definite resolution in cecal volvulus cases. The intraoperative approaches may depend on the individual case, for example, if the bowel is gangrenous; previous reports recommend resection [3]. In the present case, right hemicolectomy with side-to-side anastomosis was performed to prevent recurrence as advised in a previous report [6]. Colonoscopy is not advisable in place of surgery because of the possibility of perforation and delayed surgical management, which may cause complications and increase mortality [8].

## Conclusions

Cecal volvulus is an uncommon cause of LBO, but deadly if there is no high clinical suspicion or radiographic investigation. Taking the history of the patient into account and conducting physical examination are not specific to diagnose cecal volvulus, but CT could confirm the diagnosis to a higher percentage. In the end, surgery is still the best diagnostic and therapeutic method to treat this rare and unpleasant condition.
